# Oral and inhaled p38 MAPK inhibitors: effects on inhaled LPS challenge in healthy subjects

**DOI:** 10.1007/s00228-015-1920-1

**Published:** 2015-08-13

**Authors:** Dave Singh, Leonard Siew, Jared Christensen, Jonathan Plumb, Graham W. Clarke, Steve Greenaway, Christelle Perros-Huguet, Nick Clarke, Iain Kilty, Lisa Tan

**Affiliations:** University Of Manchester, Medicines Evaluation Unit, University Hospital of South Manchester Foundation Trust, Manchester, M23 9QZ UK; Quintiles Drug Research Unit, Respiratory and Inflammation Early Clinical Development, Quintiles Ltd, London, SE1 1YR UK; Pfizer Inc. Inflammation and Remodeling Unit, Cambridge, MA USA; Department of Cardiothoracic Pharmacology, Imperial College, National Heart and Lung Institute, London, UK

**Keywords:** p38 MAPK inhibitors, LPS challenge, Induced sputum

## Abstract

**Background:**

Inhaled LPS causes neutrophilic airway inflammation in healthy subjects. We compared the effects of p38 MAPK inhibitors and fluticasone propionate on the LPS response.

**Methods:**

Three randomised, double-blind, placebo-controlled, single dose crossover studies were performed. Active treatments were the oral p38 MAPK inhibitor PH-797804 30 mg (study 1), PH-797804 30 mg and the inhaled p38 MAPK inhibitor PF-03715455 20 mg (study 2) and inhaled fluticasone propionate 500 μg (study 3). The primary endpoint was sputum neutrophil percentage.

**Results:**

Sputum neutrophil percentage post-LPS challenge was significantly inhibited (15.1 and 15.3 % reduction) by PH-797804 compared to placebo in studies 1 and 2 (*p* = 0.0096 and 0.0001, respectively), and by PF-03715455 (8.0 % reduction, *p* = 0.031); fluticasone propionate had no effect. PH-797804 significantly inhibited the increase in inflammatory mediators (IL-6, MCP-1, MIP1β and CC16) in sputum supernatant, while PF-03715455 had no effect. PH-797804 and PF-03715455 both inhibited IL-6, MCP-1, MIP1β, CC16 and CRP levels in plasma, with PH-797804 having greater effects. Fluticasone propionate had no effect on sputum supernatant or plasma biomarkers.

**Conclusions:**

PH-797804 had the greatest impact on neutrophilic airway inflammation. Oral administration of p38 MAPK inhibitors may optimise pulmonary anti-inflammatory effects.

**Electronic supplementary material:**

The online version of this article (doi:10.1007/s00228-015-1920-1) contains supplementary material, which is available to authorized users.

## Introduction

Chronic obstructive pulmonary disease (COPD) is characterised by neutrophilic airway inflammation [1]. Neutrophils secrete pro-inflammatory mediators as well as proteases that cause tissue destruction [2]. Inhaled corticosteroids (ICS) are the most commonly used anti-inflammatory drug in COPD but have only modest clinical benefits [3] that are greatest in the subgroup of COPD patients with increased eosinophil numbers [4]. Novel drugs are needed to target neutrophilic airway inflammation in COPD.

The p38 mitogen-activated protein kinase (MAPK) signalling pathway regulates inflammatory gene expression in many different cell types by the activation of transcription factors including nuclear factor kappa-light-chain-enhancer of activated B cells (NFκB) and activating transcription factor 2 (ATF2) [5]. p38 MAPK can also contribute to inflammation by stabilising mRNAs and increasing protein translation [5]. A range of extracellular stimuli activate the p38 MAPK pathway, including toll-like receptor (TLR) agonists such as the TLR4 agonist bacterial lipopolysaccharide (LPS) [5]. The expression of activated p38 MAPK is increased in the lungs of COPD patients compared to healthy controls [6, 7], implicating this pathway in the inflammatory processes in COPD. p38 MAPK inhibitors demonstrate anti-inflammatory effects in a range of animal models of airway inflammation [8] and also reduce cytokine production from COPD alveolar macrophages, lung lymphocytes and bronchial epithelial cells in vitro [6, 9]. p38 MAPK inhibitors are currently being evaluated in clinical trials for the treatment of COPD [10–13].

Historically, orally administered p38 MAPK inhibitors have often been poorly tolerated, probably due to “off target” effects on other kinases leading to side effects such as liver toxicity [14]. More recently developed oral p38 MAPK inhibitors have greater selectivity against the p38 MAPK α and β isoforms; PH-797804 and losmapimod have been well tolerated in COPD trials up to 24-week duration [11–13]. Furthermore, PH-797804 has demonstrated a clinically significant improvement in pulmonary function [11]. However, there are still concerns about the long-term tolerability of p38 MAPK inhibitors, so an alternative strategy is to administer p38 MAPK inhibitors by inhaled delivery in order to minimise side effects by reducing systemic exposure. However, the therapeutic effectiveness of inhaled delivery for this class of drug is unknown.

The inhalation of LPS causes an influx of inflammatory cells into the airways of healthy subjects, with an increase in the proportion of neutrophils observed in induced sputum [15]. This healthy volunteer model is used as a relevant challenge model to study potential COPD treatments and has therefore been used to assess the effects of anti-inflammatory drugs on neutrophilic lung inflammation prior to performing larger studies in COPD patients [16, 17]. LPS inhalation in healthy subjects causes p38 MAPK activation in bronchial epithelial cells [18]; these cells release chemokines such as CXCL8 that promote neutrophil chemotaxis [19]. The neutrophil chemotaxis observed after LPS inhalation in healthy subjects should therefore be attenuated by drugs that inhibit p38 MAPK activation; this hypothesis has not been tested in human studies.

We have explored the effects of single doses of an orally administered and inhaled p38 MAPK inhibitor (PH-797804 and PF-03715455, respectively) on neutrophilic lung inflammation caused by LPS inhalation in healthy subjects. We also investigated the effects of a single dose of an ICS (fluticasone propionate) in this model. We present three separate clinical trials, in healthy volunteers, using inhaled LPS challenges that provide mechanistic insights into the effectiveness of oral and inhaled p38 MAPK inhibitors in a human model of neutrophilic lung inflammation that is poorly responsive to corticosteroids.

## Methods

### Subjects

Males and females of non-childbearing potential aged between 18 and 50 years were recruited. Subjects were lifelong non-smokers, or ex-smokers for >1 year with <5 pack year history. Subjects were required to have normal lung function, normal bronchial reactivity to histamine (defined as a fall in FEV1 < 20 % after inhalation of histamine at concentrations up to and including 16 mg/mL) and be able to produce an adequate sputum sample at screening with a total cell count <14 × 10^6^ cells/g comprising <70 % neutrophils and <3 % eosinophils. These sputum cell criteria were used to exclude subjects with excessive airway inflammation, which could be due to acute infection or underlying pulmonary disease. Exclusion criteria included an upper respiratory tract infection in the previous 4 weeks, or any other infection within 1 week of dosing, the presence of significant other medical conditions or clinically significant abnormalities in biochemistry or haematology blood results at screening. All subjects provided written informed consent, and the studies were approved by the local ethics committee. The studies are registered on clinicaltrials.gov: NCT02084485, NCT01314885 and NCT01364519.

### Study design

Three randomised, double-blind, placebo-controlled crossover studies were performed; the effects of a single dose of active treatment was investigated in all three studies. Study 1 was a two-way crossover study conducted at a single centre (Kings College, London) where the active treatment was PH-797804 30 mg administered orally. Studies 2 and 3 were conducted at two UK centres: Quintiles Drug Research Unit, London, and Medicines Evaluation Unit, Manchester. Study 2 was a three-way crossover study where the active treatments were a single dose of PH-797804 30 mg administered orally and a single dose of PF-03715455 20 mg by inhalation of powder inside a capsule using a single-pin monodose inhaler device (MIAT). Study 3 was a two-way crossover study where the active treatment was a single dose of inhaled fluticasone propionate 500 μg using the accuhaler™ device. The studies were performed during the following time periods: study 1, Sep 2006–Mar 2008; study 2, Jan 2011–Dec 2011; and study 3, Jul 2011–Jan 2012.

Subjects were assigned to a treatment sequence using randomised blocks by means of a computer-generated pseudo-random code generated by Pfizer. A randomisation schedule was provided to the investigator and in accordance with the randomisation numbers; the subject received the study treatment regimen assigned to the corresponding randomisation number.

The design of all three studies was similar. A baseline sputum sample was obtained within 14 days of the first study period when subjects were administered a single dose of randomised treatment. LPS challenge was administered 24 h after dosing with PH-797804/placebo in study 1; in study 2, inhaled LPS was administered on day 2 which was either 24 h after dosing with PH-797804 or 30 min after PF-03715455 as a result of the double-dummy design; in study 3, inhaled LPS was administered 30 min after dosing with fluticasone/placebo. Previous phase 1 studies in healthy subjects (unpublished) showed that the time of maximum systemic concentration (*t*_max_) for PH-797804 and PF-03715455 were at approximately 24 h and 30 min, respectively, and therefore, the LPS challenges were planned to be performed at these times. Induced sputum was obtained 6 h later to coincide with the maximal inflammatory response caused by inhaled LPS. A washout period of at least 21 days followed, with subjects returning after 14 days of the washout period for induced sputum sampling; this was performed to ensure that the total cell count, total neutrophil count and total macrophage count were within the range of −80 to +100 % of the screening values. This criterion was used to ensure that sputum cell counts after LPS challenge had returned to close to the original baseline values; this was done to reduce data variability between treatment periods. If these criteria were not met, then up to two repeat visits to obtain sputum were allowed, with at least 4 days between sputum inductions. The next randomised treatment day was scheduled at least 4 days after sputum induction and was identical to the first treatment day. The interval of 4 days between sputum inductions allowed any pro-inflammatory effect caused by sputum induction to settle before the next attempt at sampling.

Safety was assessed in these studies by physical examinations, haematology and biochemistry measurements, ECG analysis and reporting of any adverse events.

### LPS challenge

For each challenge, a 1-mg vial of lyophilised LPS (*Escherichia coli* serotype O26:B6, ref. L-2654, Sigma-Aldrich, Dorset, UK) was diluted with 4 mL of 0.9 % *w*/*v* sodium chloride. The reconstitution produced a 0.25 mg/mL solution, of which 2 mL was placed into the pre-calibrated dosimeter pot and administered via five inhalations from a breath-activated dosimeter (Mefar dosimeter MB3, Brescia, Italy). Each inhalation was performed over 3 s with a 6-s breath hold. The dosimeter delivered 12 μL for each inhalation which resulted in a total dose of 15 μg LPS.

### Induced sputum

Sputum was induced using normal saline after inhalation of salbutamol, and processing was performed using dithiothreitol (DTT) as previously described [20]. The supernatants were stored at −80 °C for later analysis, while cells were used to produce cytoslides (Cytospin 4, Shandon, Runcorn, UK) for differential cell counting and immunocytochemistry. Cytoslides for differential cell count were fixed in methanol (Sigma) and then stained with Rapi-Diff^®^ (GCC Diagnostics, Sandyhurst, UK) or Wright-modified Giemsa (Accustatin WG-18, Sigma-Aldrich); a minimum of 400 non-squamous cells were counted and differential cell counts obtained as percentage of total non-squamous cells. Cell viability was analysed by trypan blue exclusion. Cytoslides with % squamous cell counts <20 % were deemed to be of acceptable quality for differential cell counting. Unfixed cytoslides were wrapped in aluminium foil and stored frozen at −80 °C for immunocytochemistry.

#### Sputum supernatant and plasma protein biomarkers

In studies 1 and 2, sputum supernatants were analysed for interleukin 6 (IL-6), myeloperoxidase (MPO), monocyte chemotactic protein-1 (MCP-1) and macrophage inflammatory protein-1β (MIP-1 β), using electrochemiluminescent immunoassays (ECLIA) or enzyme-linked immunosorbent assays (ELISA); the manufacturers are listed in the online supplement. In study 3, sputum supernatants were analysed for IL-6, MCP-1 and MIP-1β by the same method.

Blood samples were obtained in studies 2 and 3 to obtain plasma measurements of IL-6, MCP-1, MIP1β, CC16, fibrinogen and CRP levels; pre-dose and 6 h post-LPS samples were used for statistical analysis. This coincided with the timings of sputum measurements of inflammation biomarkers.

#### Immunocytochemistry

Frozen cytospins created from sputum cells were analysed for phosphorylated-Heat Shock Protein 27 (phospho-HSP27) and phospho-p38 expression in sputum macrophages. The methods are fully described in the online supplement. Phospho-p38 and phospho-HSP27 immunoreactivity is presented as percentage of the macrophage population. All analyses were carried out by blinded observers.

#### Pharmacokinetics

Blood samples were collected at 0 h and around the time of *t*_max_ and at the time of sputum induction (23 and 30.5 h post dose for PH-797804 and 1.5 h and 6.5 h post-dose for PF-03715455) to provide plasma for pharmacokinetic analysis using previously validated analytical methods. Sparse sampling was employed to provide data at C_max_ to confirm drug levels of PH-797804, PF-03715455 and fluticasone propionate relative to LPS challenge. PF-03715455, PH-797804 and fluticasone propionate plasma concentrations were analysed using high-performance liquid chromatography tandem mass spectrometric methods at Advion BioServices, Inc. (Ithaca, NY, USA), PPD (Richmond, VA, USA) and York Bioanalytical Solutions (York, UK), respectively; the lower limits of quantification were 10 pg/mL, 0.1 ng/mL and 3 pg/mL, respectively.

### Statistical methods

Sputum neutrophil percentage was the primary endpoint for all three studies. The neutrophil percentage was modelled using a mixed effects analysis of covariance (ANCOVA) model appropriate for a two-period, two-treatment or three-period, three-treatment crossover design. The fixed effects in the model were treatment and period. Subjects were modelled as a random effect. Additionally, the period-specific baseline was modelled as a covariate. This model was used for all sputum inflammatory markers, including the immunohistocytochemistry, where cell counts were log transformed before analyzing the data and percent differentials were not transformed. This model used all available information from the subject visits to estimate treatment effects; the data for subjects who were randomised but did not complete the study were still included in the modelling. Ninety percent confidence intervals of the difference (non-transformed data) or the ratio (log-transformed data) were constructed based on the ANCOVA model.

An additional non-informative Bayesian analysis was proposed for studies 2 and 3, but only the frequentist analysis is presented here. Bayesian analyses offer the advantage of easily combining results across multiple studies. A Bayesian analysis was proposed to aggregate data across these three studies in a more coherent framework. The frequentist analysis was pre-specified and is presented in this paper to limit conclusions to only the data in a single study. The non-informative Bayesian analysis and the frequentist analysis gave similar results and showed no difference in interpretation or conclusion, as expected based on the statistical theory behind these analyses.

Systemic biomarkers were analysed using a similar mixed effects model; these models also adjusted for pre-dose baseline values, which were obtained 24 h prior to LPS challenge in study 2 and 30 min prior to LPS challenge in study 3.

The three studies have all been powered at least at 80 % with an alpha of 0.05 (study 1 and 2) or 0.10 (study 3). The sample size calculations for study 1 were based on previous LPS challenge data in healthy volunteers. A maximum sample size of 24 was estimated to be sufficient based on the standard deviation observed previously for measurements including sputum neutrophil percentage, and an interim analysis after 12 subjects was planned to re-evaluate variability within the study and re-estimate the sample size. Study 1 was stopped at the interim analysis, as the variability observed for neutrophil percentage was lower than anticipated. Study 1 was used to estimate the within subject variability (SD) for study 2; standard deviation for sputum neutrophil percentage of 19 % and an effect size of 15 % was assumed, giving a sample size of 18. The variability from study 2 was used to further refine the estimate of within-subject variability for study 3, SD = 15, with an assumed effect size of 15 % which gave a sample size of 12.

## Results

**Fig. 1:**
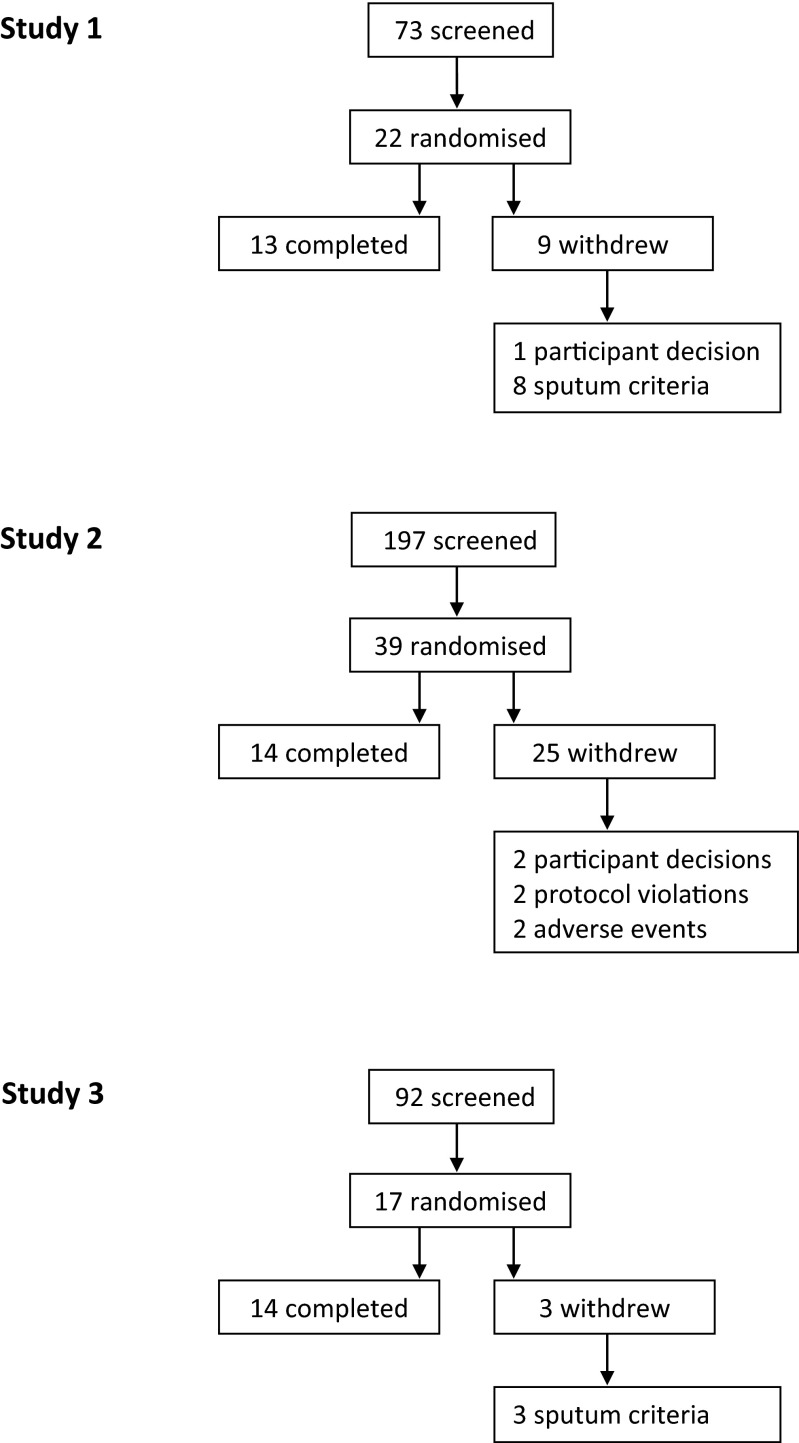
Flow diagram of patient numbers. This shows the number of subjects screened, randomised, withdrawn and completed for each study

The number of subjects randomised and who completed the studies is shown in Fig. 1. The majority of screen failures were due to failure to provide an adequate sputum sample according to the inclusion criteria. The next most common reason was the discovery of significant medical conditions or abnormal blood results at screening. The demographic details of the subjects randomised are shown in the online supplement. In study 1, 22 male subjects were randomised (mean age 34 years), of which 13 completed both treatment periods. In study 2, 39 male subjects were randomised (mean age 28 years), of which 14 completed all three treatment periods. In study 3, 17 male subjects were randomised (mean age 28 years), of which 14 completed both treatment periods. The major reason for non-completion in all three studies was failure to meet the sputum reproducibility criteria before entering the next treatment period. PH-797804 and PF-03715455 were safe and generally well tolerated; a summary of the number of subjects experiencing adverse events by MedDRA System Organ Class is shown in the online supplement. Two subjects suffered with AEs requiring withdrawal in study 2; one reported malaise after placebo treatment, and one developed cellulitis 5 days after being treated with PF-03715455; further details of this event are in the online supplement.

### Sputum neutrophil percentage

Table 1 displays the mean sputum neutrophil percentage in all three studies at baseline and 6 h following LPS challenge. The baseline neutrophil percentages were similar across the three studies. Similar values were observed within each study prior to each treatment period (approximately 41–44 %), demonstrating no carry-over effect of LPS challenge on neutrophil counts. There were similar increases in mean sputum neutrophil percentage in the placebo period after LPS challenge (which will be referred to as the LPS response from now) across the three studies; modelled LPS responses of approximately 30 % were observed.

**Table 1:** Mean sputum neutrophil percentage at baseline and 6 h post-LPS challenge

Study	Treatment	Period	Number	Mean (SD)
1	Placebo	Baseline	18	44.1 (17.0)
		6 h post-LPS	17	74.0 (17.6)
	PH-797804 30 mg	Baseline	17	42.1 (17.8)
		6 h post-LPS	14	56.4 (24.0)
2	Placebo	Baseline	27	41.5 (17.6)
		6 h post-LPS	25	76.2 (12.1)
	PF-03715455 20 mg	Baseline	25	44.9 (15.6)
		6 h post-LPS	24	66.4 (14.0)
	PH-797804 30 mg	Baseline	22	42.0 (13.4)
		6 h post-LPS	22	60.4 (17.9)
3	Placebo	Baseline	14	41.6 (12.1)
		6 h post-LPS	11	74.1 (12.2)
	Fluticasone 500 μg	Baseline	15	43.2 (15.2)
		6 h post-LPS	15	70.0 (12.9)

*SD* standard deviation

**Fig. 2:**
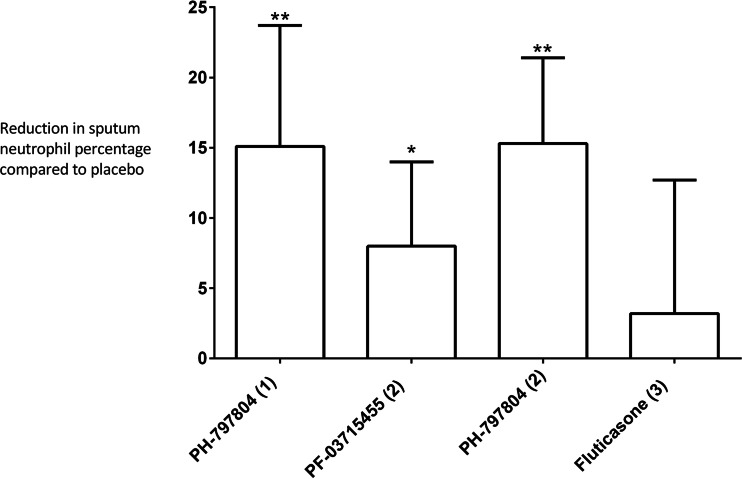
Inhibition of LPS induced sputum neutrophil percentage. The reduction in sputum neutrophil percentage caused by active treatments compared to placebo are shown; *bars* are mean difference and error bars are 90 % CI (*p < 0.05 and **p < 0.01 from ANCOVA model)

There was a statistically significant inhibition of the sputum neutrophil percentage post-LPS challenge caused by PH-797804 compared to placebo in studies 1 and 2 (*p* = 0.0096 and 0.0001, respectively), with 15.1 and 15.3 % reduction in mean sputum neutrophil percentage (Fig. 2), representing approximately 50 % attenuation of the modelled LPS response. Study 2 also showed significant inhibition of sputum neutrophil percentage post LPS challenge after administration of PF-03715455 of 8.0 % which was approximately 25 % attenuation of the modelled LPS response (*p* = 0.031). Sputum neutrophil percentage post-LPS challenge was not changed significantly by fluticasone propionate (*p* = 0.55).

#### Secondary endpoint sputum measurements

The total neutrophil cell count was increased by LPS challenge in the placebo treatment period in all three studies (data shown in online supplement). PH-797804 significantly inhibited the total neutrophil count in sputum compared to placebo in studies 1 and 2. PF-03715455 and fluticasone propionate had no effects on neutrophil counts. In study 2, both PH-797804 and PF-03715455 significantly attenuated the reduction in sputum macrophage percentage compared to placebo; this is compatible with the inhibitory effect of these drugs on the increase in sputum neutrophil percentage after LPS challenge (data shown in online supplement).

**Fig. 3:**
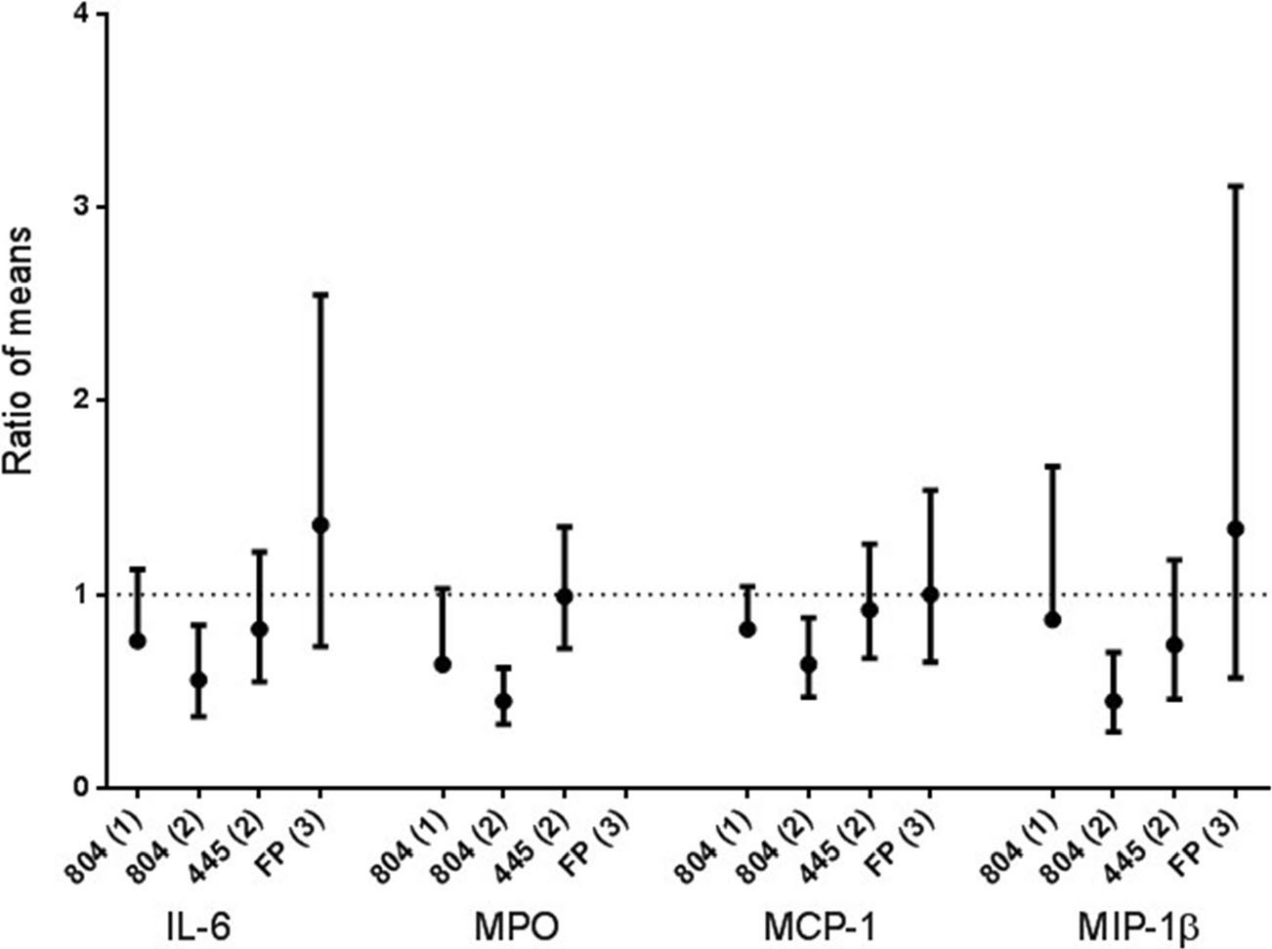
Sputum cytokine data. The ratio of means (with bars showing 90 % CI) of active treatment compared to placebo is shown. *Numbers in brackets* on *x*-axis denote study number, i.e., study 1, 2 or 3. Drug abbreviations are as follows: 804 = 797804, 445 = 03715445, *FP* fluticasone propionate

Sputum supernatant cytokine levels were generally increased by LPS challenge in the placebo treatment period in all three studies (data shown in online supplement). In study 1, PH-797804 did not significantly inhibit cytokine levels compared to placebo, but there was evidence of trends towards significance for MCP-1 and MPO (see Fig. 3). In study 2, PH-797804 significantly reduced IL-6, MPO, MCP-1 and MIP-1β levels compared to placebo. PF-03715455 and fluticasone propionate had no effects on sputum cytokine measurements.

### Systemic biomarkers

**Fig. 4:**
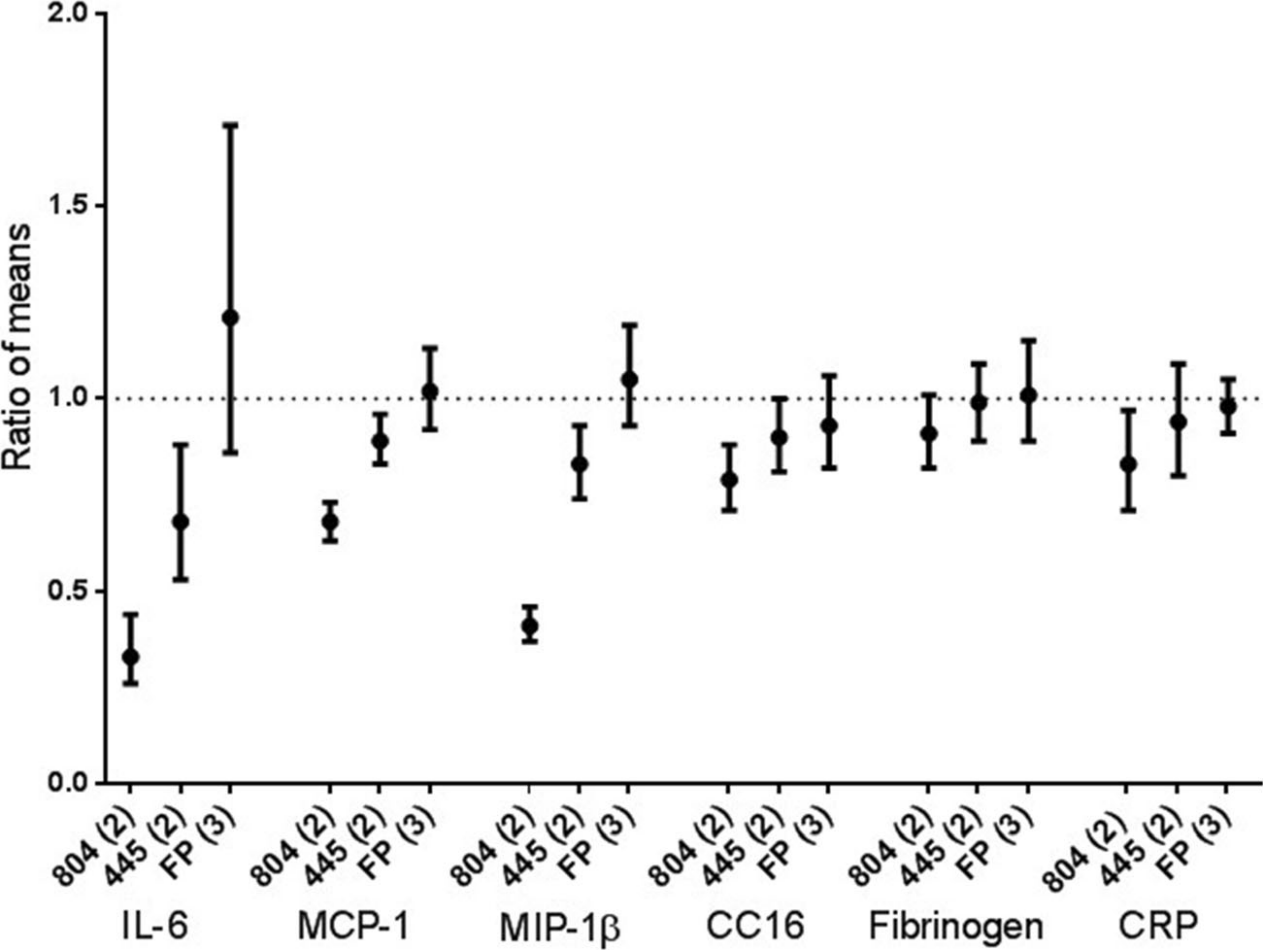
Systemic biomarker data. The ratio of means (with bars showing 90 % CI) of active treatment compared to placebo is shown. *Numbers in brackets* on *x*-axis denote study number, i.e., study 2 or 3. Drug abbreviations are as follows: 804 = 797804, 445 = 03715445, *FP* fluticasone propionate

PH-797804 caused statistically significant reductions in IL-6, MIP1β, MCP-1, CC16 and CRP levels compared to placebo at 6 h post-LPS challenge (see Fig. 4 for ratio of means; numerical values at each time point are shown in online supplement). When comparing PF-03715455 to placebo, statistically significant reductions in IL-6, MCP-1, MIP1β and CC16 were observed. PH-797804 showed a greater numerical effect on these biomarkers than PF-03715455. Fluticasone propionate had no effect on this set of systemic biomarkers compared to placebo.

### Sputum immunohistochemistry

Immunohistochemistry performed on samples in study 2 showed that phospho-P38 and phospho-HSP27 expression in sputum cells was restricted to macrophages, with little or no expression in neutrophils; we have previously reported this finding in healthy subjects and COPD patients [6]. LPS challenge did not increase the percentage of macrophages expressing phospho-P38 or phospho-HSP27 compared to baseline in the placebo treatment period (see Table 2). PH-797804 had no effect on the percentage of macrophages expressing phospho-P38 and a non-significant difference on phospho-HSP27 after LPS challenge. In contrast, PF-03715455 significantly reduced the percentage of macrophages expressing phospho-P38 and phospho-HSP27; these decreases correspond to an attenuation of the baseline measurements of approximately 45–50 %.

**Table 2:** Inhibition of phospho-p38 and phospho-HSP27 expression in sputum samples in study 2

			Comparison vs placebo		
	Baseline	Post-LPS	Mean % inhibition	*P* value	Confidence limits
Phospho-p38 expression					
PF-03715455	51.30 %	25.03 %	−14.59	0.0022	−21.85; −7.34
PH-797804	40.76 %	38.00 %	−1.67	0.6958	−8.89; 5.55
Placebo	39.01 %	37.97 %	NA	NA	NA
Phospho-HSP27 expression					
PF-03715455	47.88 %	26.89 %	−24.01	0.0014	−35.53; −12.49
PH-797804	49.34 %	42.27 %	−5.81	0.4060	−17.53; 5.90
Placebo	47.16 %	46.36 %	NA	NA	NA

Samples post-LPS challeng were analysed. The mean percentage of macrophages expressing the protein is shown, with mean inhibition and 90 % confidence intervals

### Pharmacokinetics

The systemic drug concentrations at the expected C_max_, for each study, are shown in Table 3. As expected, the plasma C_max_ for orally administered PH-797804 was greater than the inhaled drugs. Sputum supernatants, from study 2, were also analysed for drug concentrations, for the ten subjects that had received all three treatments; the geometric mean sputum concentration of PF-03715455 after receiving an inhaled dose was 20 nM (range 4–73 nM), while the geometric mean sputum concentration of PH-797804 after receiving an oral dose was 4 nM (range 2–7 nM).

**Table 3:** Plasma pharmacokinetics

Study	Treatment	Number	Units	1.5 h	6.5 h	23 h	30.5 h
1	PH-797804 (30 mg)	17	ng/mL	–	–	38.9 (7.4)	37.8 (8.0)
2	PF-03715455 (20 mg)	26	pg/mL	472.6 (116.4)	110.6 (29.7)	–	–
	PH-797804 (30 mg)	23	ng/mL	–	–	56.8 (18.7)	49.2 (14.7)
3	Fluticasone (500 μg)	15	pg/mL	56.0 (29.4)	37.31 (15.1)	–	–

The selected time points used in each study are shown

“–” no samples were taken

## Discussion

The p38 MAPK inhibitors PH-797804 and PF-03715455 both reduced neutrophilic airway inflammation after LPS challenge in healthy subjects. A consistent effect of PH-797804 on sputum neutrophils was observed in two different studies. In contrast, inhaled fluticasone propionate at a dose of 500 μg had no effect. Interestingly, the orally administered p38 MAPK inhibitor had a greater effect on sputum neutrophils and supernatant cytokines than the inhaled drug. Inhaled delivery is often favoured for respiratory drugs, as it increases lung compared to systemic exposure with the aim of increasing therapeutic pulmonary effects relative to systemic side effects. However, it appears that increased systemic exposure of orally administered PH-797804 provided superior pulmonary anti-inflammatory effects in a human inhaled LPS challenge model that is poorly responsive to corticosteroids.

PH-797804 and PF-03715455 both suppressed pulmonary and systemic biomarkers of inflammation, with greater effects observed for PH-797804. PH-797804 had a significant effect on sputum neutrophils in study 1. Study 2 confirmed a similar effect size, indicating that PH-797804 had a repeatable effect on the reduction of neutrophilic airway inflammation. Study 2 showed that PH-797804 significantly inhibited sputum cytokines, while only trends to significance were observed in study 1; the differences between studies are likely to be due to variability in relatively limited sample sizes.

The effects of PH-797804 and PF-03715455 on systemic inflammation could either be attributed to the activity of the compound absorbed into the systemic circulation, or to the inhibition of pulmonary inflammation leading to a reduced systemic response to inhaled LPS. The plasma concentrations of PF-03715455 at the time of LPS challenge were below the IC_50_ value for the drug (enzyme IC_50_ 5–50 pM), suggesting minimal systemic activity. In our view, the anti-inflammatory effects of PF-03715455 on systemic biomarkers were mainly due to the effects of the compound in the lung. In contrast, the increased effects of PH-797804 compared to PF-03715455 in this LPS challenge model are probably due to greater systemic exposure of the oral compound limiting inflammatory cell recruitment into the lungs and also reducing systemic inflammation.

The greater effect of PF-03715455 on sputum biomarkers of p38 MAPK activation may be explained by the fact that the geometric mean sputum concentration of PF-03715455 was 20 nM, which is above the enzyme IC_50_ for PF-03715455 (5–50 pM). The geometric mean concentration of PH-797804 in sputum supernatant was 4 nM, which is similar to the IC_50_ for PH-797804 (3 nM).

A bronchoscopy study has shown that inhaled LPS challenge in healthy subjects activates p38 MAPK in bronchial epithelial cells [18]. These cells release neutrophil chemoattractants such as CXCL8 after p38 MAPK activation [6, 21]. Epithelial cells may be key controllers of the inflammatory response to LPS in the airways. Both PH-797804 and PF-03715455 significantly inhibit IL-8 production from epithelial cells [Pfizer, data on file] and may therefore reduce neutrophil recruitment to the lung. Cytokines measured in induced sputum are secreted either by cells within the airway lumen or by airway epithelial cells. The lack of effect of PF-03715455 on sputum supernatant cytokines, in contrast to PF-03715455 inhibition of sputum macrophage p38 MAPK activation, suggests that the cytokines measurable in induced sputum were predominantly derived from epithelial secretion.

We observed no upregulation of phospho-p38 or phospho-HSP27 expression at 6 h after LPS challenge. This was perhaps surprising, as LPS challenge increases bronchial epithelial p38 MAPK activation [18], and LPS stimulation is known to upregulate phospho-P38 expression in human alveolar macrophages [9]. However, the phospho-p38 expression in LPS-stimulated alveolar macrophages is transient [9], and it is possible that the 6 h sampling point after LPS challenge was not optimal for this measurement. Nevertheless, measuring p38 MAPK activation in sputum samples provided insights into the relative effects of the drugs studied on cells that are resident within the airway lumen.

It has been shown that PH-797804 improves FEV_1_ by 93 mL in COPD patients treated for 6 weeks [12]. The current study shows that PH-797804 is capable of reducing airway neutrophil numbers and airway inflammatory biomarkers in healthy subjects after LPS challenge, providing an insight into the possible mechanisms by which this compound exerts anti-inflammatory effects in the airways. The oral p38 MAPK inhibitor losmapimod failed to reduce sputum neutrophil counts in COPD patients after 12-week treatment [11]; the investigators suggested that this may be due to a technical failure of the study regarding sputum analysis. We studied PH-797804 in young healthy volunteers, as it is safer to conduct LPS challenges in these subjects compared to older individuals or even patients with COPD [22]. The reduction in airway neutrophil numbers in this healthy volunteer challenge model differs from studying drug effects in COPD patients with chronic neutrophilic airway inflammation; nevertheless, our results support the case for further studies to investigate the effects of this drug on sputum neutrophil numbers in COPD patients.

It has been reported that 6-day treatment with the oral corticosteroid prednisolone had no effect on airway inflammation in an LPS challenge study but suppressed plasma CRP levels [16]. The lack of effect of inhaled fluticasone propionate on airway inflammation is consistent with these previous findings. Given the lack of effect of oral prednisolone on airway inflammation in this model, we speculate that a higher dose of inhaled fluticasone propionate, or given for a longer duration, also would not have inhibited airway inflammation in this acute model of lung neutrophilia, but this remains to be studied. It has been reported that a single dose of inhaled fluticasone propionate can significantly inhibit the effects of bronchial challenges in asthma [23]; notably, a single dose given 30 min before allergen challenge had a similar effect compared to 2 weeks prior treatment [24]. This supports the use of a single dose of fluticasone propionate in challenge models.

We started the LPS challenges at the time of maximum plasma concentration of the drugs. As sputum samples were taken at 6 h later, this gave the drugs a time window after maximal plasma concentration to exert anti-inflammatory effects. Different factors will influence the effects of a single drug dose in the lungs, such as potency, plasma half-life (which will influence total systemic exposure) and lung retention. This study attempted to capture the maximum effects of the drugs based on the known maximal plasma concentration, which were achieved at very different times (longer for PH-797804, with an estimated *t*_max_ at approximately 23 h). However, the effects of these drugs are dependent on many factors, including the timing of the LPS challenge and the number of drug doses administered before challenge.

Studies 1 and 3 (two-way crossover studies) had a combined completion rate of 69 % (27 out of 39 subjects), while in study 2 (three-way crossover), the completion was 36 % (14 out of 39 subjects). This indicates a failure to meet the sputum criteria of approximately 30 % occurs before each crossover period. We set sputum criteria to limit the variability of the primary endpoint; the practical drawback was the subsequent drop-out rate. The data from patients who did not complete the study were included in the statistical analysis, thus limiting the bias potentially caused by only evaluating patients who completed the study. However, more data were available for patients who completed the study, and the effect sizes reported here are applicable to healthy volunteers who met the sputum criteria specified; these drug effects may vary with the sputum criteria used.

The effects of PH797804 in studies 1 and 2 were very similar. This indicates a reasonable degree of comparability between the studies that used similar designs, and the same inclusion criteria to recruit a homogenous volunteer group (healthy males). Furthermore, many of our key findings come from study 2, where both p38 MAPK inhibitors were compared to placebo. Ideally, one would like to study the three active treatments reported here within the same crossover design; this would be a four-way crossover including a placebo. However, the practical difficulties in terms of subject drop outs that we experienced during a three-way crossover would make a longer study extremely difficult to complete.

P38 MAPK inhibitors have been in clinical development for many years for a range of inflammatory diseases. Unfortunately, many orally administered P38 MAPK inhibitors have been poorly tolerated due to side effects. The development of inhaled therapies may optimise the therapeutic index by increased pulmonary versus systemic exposure. We have used a model of acute neutrophilic airway inflammation to compare the effects of an oral and inhaled p38 MAPK inhibitor, as well as an inhaled corticosteroid. The LPS challenge model mimics some aspects of acute exacerbations of COPD, which are often caused by bacterial infections leading to increased TLR signalling. PH-797804 and PF-03715455 both reduced airway and systemic inflammation after LPS inhalation, and therefore show promise as potential new anti-inflammatory treatments of neutrophilic airways diseases such as COPD.

## Electronic supplementary material

ESM 1 (DOCX 27 kb)
