# Supplementary material for: Oral and inhaled p38 MAPK inhibitors: effects on inhaled LPS challenge in healthy subjects
Source: Eur J Clin Pharmacol. 2015 Aug 13;71(10):1175–84. doi: 10.1007/s00228-015-1920-1 (PMC4564450; doi:10.1007/s00228-015-1920-1)
Supplement: Supplementary file 1 — (DOCX 27 kb) [file 228_2015_1920_MOESM1_ESM.docx]

**ON-LINE SUPPLEMENT**

**Assay Manufacturers Information**

Assay were performed at SGS-Cephac Europe, using the kits listed in the table

| **Analytes** | **Matrix** | 1. **Assay Test Kit** | **Method of Detection** |
| --- | --- | --- | --- |
| IL-6, MCP-1 and MIP-1beta | Human Plasma | Study 2:  Meso Scale Discovery® (MSD®,Gaithersburg, MD, USA), Multi-Spot® 96 4-Spot Custom 4-Plex, Catalog No. N45CA-1 and Catalog No. N45IA-1  Study 3:  Meso Scale Discovery® (MSD®), Multi-Spot® 96 4-Spot Custom 4-Plex, Catalog No. N45IA-1 | ECLIA |
| CRP | Human Plasma | Studies 2 & 3:  MSD® 96-Well Multi-Array® CRP Assay, Catalog No. K151EPC | ECLIA |
| CC16 | Human Plasma | Studies 2 & 3:  BioVendor (Heidelberg,  Germany), Human Clara Cell Protein ELISA, Catalog No. RD191022200 | ELISA |
| Fibrinogen | Human Plasma | Studies 2 & 3:  GenWay (San Diego, CA, USA), Human Fibrinogen ELISA Kit, Catalog No. 40-374-130105 | ELISA |
| IL-6, MCP-1 and MIP-1beta | Human Sputum | Study 2:  MSD® Multi-Spot® 96 4-Spot Custom 3-Plex, Catalog No. N45CA-1  Study 3:  MSD® Multi-Spot® 96 4-Spot Custom 4-Plex, Catalog No. N45IA-1  Study 1:  Human extracellular protein buffer reagent kit fromBiosource International (Paisley, UK), Ref. LHB0001,Lot no. 1383553.  MIP-1beta kit, Ref. LHC1051, Lot no. 1387254.  MCP-1 kit, Ref. LHC1011, Lot no. 1368914.  IL-6 kit, Ref. LHC0061, Lots no. 1376502 and1394569. | ECLIA  ECLIA  Multiplex (Luminex) |
| MPO | Human Sputum | Study 2:  MSD® Multi-Array®Assay System Human  Myeloperoxidase (MPO) Assay Kit, Catalog  No. K151EEC  Study 1:  MPO ELISA kit from Immundiagnostik AG (Bensheim, Germany),ref. K6630 (batches no. 070104, 070307 and 070814A) | ECLIA  UV Absorbance |

**Subject Demography**

|  | Study 1 | Study 2 | Study 3 |
| --- | --- | --- | --- |
| No. of subjects | 22 | 39 | 17 |
| Age | 34.0 (9.1) | 28.4 (7.9) | 27.7 (6.9) |
| Weight | 80.6 (10.9) | 79.7 (9.0) | 83.2 (11.1) |
| BMI | 26.3 (3.0) | 24.8 (2.9) | 25.7 (3.1) |

**Immunocytochemistry**

Frozen cytospins created from sputum cells were thawed and the cells were fixed in 4% paraformaldehyde in PBS for 10 minutes at room temperature, followed by blocking in normal serum. Cytospins were then incubated with the following primary antibodies overnight at 4 °C; anti-human phosphorylated-p38 (phospho-p38) MAPK (Thr180/Tyr182) antibody (Cat No. 9211S, New England Biolabs, Herts, UK) or phosphorylated-Heat Shock Protein 27 (phospho-HSP27 [Cat No. S82, New England Biolabs]) diluted 1:50 in 1.5% normal serum with 0.5% Triton X-100 (Sigma-Aldrich). Endogenous peroxidase was quenched by incubating cytospins in 3% H_2_O_2_ in methanol (0.3% for phospho-p38). Phospho-p38 and phospho-HSP27 were detected using biotinylated goat anti-rabbit IgG secondary antibody (Vector Labs, Peterborough, UK) in conjunction with an avidin–biotin peroxidase complex (Vector Labs) and 3,3’-diaminobenzidine (DAB) substrate. Cell nuclei were counterstained with Gill’s haematoxylin (Sigma-Aldrich). Omission of primary antibody in staining protocol was used as a negative control.

Digital micrographs were obtained through the use of a Nikon Eclipse 80i microscope (Nikon UK Ltd, Surrey, UK) equipped with a QImaging digital camera (Media cybernetics, Marlow, UK) and ImageProPlus 5.1 software (Media Cybernetics). Sputum macrophages (determined by morphology) were analysed for phospho-p38 and phospho-HSP27 expression following single label immunohistochemistry utilising the ImagePro Plus image analysis software; at least 200 macrophages per cytospin were counted. Phospho-p38 and phospho-HSP27 immunoreactivity was presented as percentage of the macrophage population. All analysis was carried out by blinded observers.

**Sputum Cell Count Data.**

Mean values are shown; The baseline values are unadjusted means, while post LPS values are from the ANCOVA model that includes adjustment for baseline. * Data shown for macrophage % is difference between the active treatment mean and the placebo mean; including upper and lower limits of the CI of the difference (i.e. not ratios)

|  | Study | Compound | Active Treatment | | Placebo | | | Ratio of Means | Lower Limit of Ratio | Upper Limit of Ratio |
| --- | --- | --- | --- | --- | --- | --- | --- | --- | --- | --- |
|  |  |  | Baseline | Post LPS | Baseline | | Post LPS |  |  |  |
| Total Cell Count (x10^6^/g) | 1 | PH-797804 | - | - | - | | - | - | - | - |
|  | 2 | PH-797804 | 2.45 | 2.02 | 2.28 | | 3.76 | 0.54 | 0.36 | 0.80 |
|  | 2 | PF-03715445 | 1.98 | 3.83 | 2.28 | | 3.76 | 1.02 | 0.69 | 1.51 |
|  | 3 | Fluticasone | 2.78 | 3.02 | 3.41 | | 3.34 | 0.90 | 0.50 | 1.65 |
|  | | | | | | | | | |  |
| Total Neutrophil Count (x10^6^/g) | 1 | PH-797804 | 0.6 | 0.66 | 0.54 | | 1.40 | 0.47 | - | 0.83 |
|  | 2 | PH-797804 | 1.11 | 1.17 | 1.08 | | 2.85 | 0.41 | 0.26 | 0.65 |
|  | 2 | PF-03715445 | 0.93 | 2.43 | 1.08 | | 2.85 | 0.85 | 0.54 | 1.34 |
|  | 3 | Fluticasone | 1.27 | 2.04 | 1.52 | | 3.09 | 0.66 | 0.34 | 1.27 |
|  | | | | | | | | | |  |
| Total Macrophage Count (x10^6^/g) | 1 | PH-797804 | 0.84 | 0.47 | 0.78 | | 0.37 | 1.27 | - | 72.6 |
|  | 2 | PH-797804 | 1.26 | 0.62 | 1.12 | | 0.67 | 0.92 | 0.63 | 1.34 |
|  | 2 | PF-03715445 | 0.99 | 1.09 | 1.12 | | 0.67 | 1.62 | 1.12 | 2.36 |
|  | 3 | Fluticasone | 1.44 | 0.82 | 1.81 | | 0.87 | 0.93 | 0.51 | 1.7 |
|  | | | | | | | | | |  |
| Macrophage Differential (%)* | 1 | PH-797804 | - | - | | - | - | - | - | - |
|  | 2 | PH-797804 | 54.59 | 36.39 | | 55.05 | 22.81 | 13.58 | 7.33 | 19.83 |
|  | 2 | PF-03715445 | 52.32 | 30.78 | | 55.05 | 22.81 | 7.97 | 1.88 | 14.06 |
|  | 3 | Fluticasone | 53.65 | 27.53 | | 56.27 | 25.49 | 2.04 | -6.37 | 10.45 |

**On-line: Sputum Cytokine Data.** Mean values are shown for active and placebo at both pre and post baseline measurements

|  | **Study** | **Compound** | **Active Treatment** | | **Placebo** | |
| --- | --- | --- | --- | --- | --- | --- |
|  |  |  | Baseline | Post LPS | Baseline | Post LPS |
| **IL-6 (pg/mL)** | 1 | PH-797804 | 18.06 | 29.66 | 24.00 | 84.19 |
|  | 2 | PH-797804 | 75.96 | 69.02 | 58.79 | 131.84 |
|  | 2 | PF-03715445 | 61.47 | 91.67 | 58.79 | 131.84 |
|  | 3 | Fluticasone | 121.28 | 200.97 | 121.31 | 143.89 |
|  | | | | | | |
| **MPO (ng/mL)** | 1 | PH-797804 | 596.88 | 669.06 | 622.17 | 1007.70 |
|  | 2 | PH-797804 | 456.42 | 485.28 | 617.09 | 970.68 |
|  | 2 | PF-03715445 | 394.03 | 937.04 | 617.09 | 970.68 |
|  | 3 | Fluticasone | ND | ND | ND | ND |
|  | | | | | | |
| **MCP-1 (pg/mL)** | 1 | PH-797804 | 147.06 | 155.07 | 166.89 | 219.11 |
|  | 2 | PH-797804 | 86.75 | 133.41 | 84.89 | 133.98 |
|  | 2 | PF-03715445 | 94.91 | 115.38 | 84.89 | 133.98 |
|  | 3 | Fluticasone | 148.41 | 133.79 | 173.34 | 122.58 |
|  | | | | | | |
| **MIP-1β (pg/mL)** | 1 | PH-797804 | 108.01 | 330.54 | 157.51 | 496.31 |
|  | 2 | PH-797804 | 172.6 | 609.10 | 227.12 | 975.88 |
|  | 2 | PF-03715445 | 161.18 | 396.35 | 227.12 | 975.88 |
|  | 3 | Fluticasone | 165.88 | 1157.87 | 203.68 | 779.15 |

**On-line table: Systemic Cytokine Data.** Mean values are shown for active and placebo at both pre and post baseline measurements

|  | **Study** | **Compound** | **Active Treatment** | | **Placebo** | |
| --- | --- | --- | --- | --- | --- | --- |
|  |  |  | **Baseline** | **6 hrs Post LPS** | **Baseline** | **6 hrs Post LPS** |
| **IL-6 (pg/mL)** | 2 | PH-797804 | 3.67 | 5.47 | 11.14 | 26.38 |
|  | 2 | PF-03715445 | 4.34 | 14.43 | 11.14 | 26.38 |
|  | 3 | Fluticasone | 4.77 | 23.30 | 5.89 | 22.33 |
|  | | | | | | |
| **MCP-1 (pg/mL**) | 2 | PH-797804 | 349.6 | 261.50 | 352 | 394.70 |
|  | 2 | PF-03715445 | 339.3 | 340.30 | 352 | 394.70 |
|  | 3 | Fluticasone | 338.1 | 401.70 | 342.7 | 390.60 |
|  | | | | | | |
| **MIP-1β (pg/mL)** | 2 | PH-797804 | 60.8 | 33.82 | 62.81 | 84.34 |
|  | 2 | PF-03715445 | 63.35 | 71.55 | 62.81 | 84.34 |
|  | 3 | Fluticasone | 64.47 | 76.95 | 60.51 | 69.79 |
|  | | | | | | |
| **CC16**  **(ng/mL)** | 2 | PH-797804 | 6.94 | 9.34 | 6.14 | 10.55 |
|  | 2 | PF-03715445 | 6.75 | 10.19 | 6.14 | 10.56 |
|  | 3 | Fluticasone | 6.43 | 10.51 | 6.62 | 11.82 |
|  | | | | | | |
| **Fibrinogen**  **(mcg/mL)** | 2 | PH-797804 | 2243.4 | 2172.70 | 2114.4 | 2331.90 |
|  | 2 | PF-03715445 | 2284 | 2340.40 | 2114.4 | 2331.90 |
|  | 3 | Fluticasone | 1855.3 | 2058.00 | 1892.1 | 1998.60 |
|  | | | | | | |
| **CRP**  **(ng/mL)** | 2 | PH-797804 | 700.1 | 462.10 | 763.3 | 618.00 |
|  | 2 | PF-03715445 | 646.6 | 475.90 | 763.3 | 618.00 |
|  | 3 | Fluticasone | 783.7 | 708.10 | 467 | 465.00 |

**Adverse Events**

Summary of Number ofSubjects Experiencing Treatment EmergentAdverse Events by MedDRA System Organ Class

**Study 1**

|  | PH-797804 30mg  (n=17) | Placebo  (n=18) |
| --- | --- | --- |
| Eye disorders | 1 | 0 |
| Gastrointestinal disorders | 0 | 1 |
| General disorders and administration site conditions | 1 | 1 |
| Infections and infestations | 1 | 2 |
| Injury, poisoning and procedural complications | 1 | 0 |
| Investigation (raised blood potassium) | 0 | 1 |
| Musculoskeletal and connective tissue disorders | 1 | 1 |
| Nervous system disorders | 2 | 3 |
| Psychiatric disorders* | 1 | 0 |
| Respiratory, thoracic and mediastinal disorders | 0 | 1 |
| Skin and subcutaneous tissue disorders | 0 | 3 |

- This psychiatric event was insomnia lasting 1 hr

**Study 2**

|  | PF-03715455 20mg (n=27) | PH-797804 30mg (n=23) | Placebo (n=27) |
| --- | --- | --- | --- |
| Eye Disorders | 0 | 1 | 0 |
| Gastrointestinal Disorders | 2 | 0 | 1 |
| General Disorders and Administration Site Conditions | 3 | 3 | 3 |
| Immune system disorders | 0 | 0 | 1 |
| Infections and Infestations | 5 | 2 | 5 |
| Injury, Poisoning and Procedural Complications | 0 | 1 | 1 |
| Musculoskeletal and Connective tissue disorders | 0 | 0 | 2 |
| Nervous system disorders | 5 | 8 | 2 |
| Psychiatric disorders* | 1 | 2 | 0 |
| Respiratory, thoracic and mediastinal disorders | 2 | 3 | 4 |
| Skin and subcutaneous tissue disorders | 1 | 0 | 0 |
| Vascular disorders | 1 | 0 | 0 |

* These psychiatric events were hangover (PF-03715455), and loss of libido and insomnia (both PH-797804)

One patient treated with PF-03715455 developed cellulitis 5 days after dosing. The subject was successfully treated with intravenous antibiotics then oral antibiotics. The cellulitis resolved 7 days following discharge from hospital. The subject had previously been treated with oral antibiotics for infected insect bites. The investigator did not consider that the event was related to PF-03715455.

**Study 3**

|  | Fluticasone Proprionate 500ug (n=15) | Placebo  (n=16) |
| --- | --- | --- |
| Ear and labyrinth disorders | 1 | 0 |
| Gastrointestinal disorders | 1 | 0 |
| General disorders and administration site conditions | 2 | 1 |
| Infections and infestations | 1 | 2 |
| Injury, poisoning and procedural complications | 0 | 2 |
| Respiratory, thoracic and mediastinal disorders | 2 | 1 |
